# Flavobacterium aerium sp. nov., a bacterium isolated from the air of the Icelandic volcanic island Surtsey

**DOI:** 10.1099/ijsem.0.006647

**Published:** 2025-01-23

**Authors:** Aurélien Daussin, Pauline Vannier, Marine Ménager, Émilien Mater, Viggó Þór Marteinsson

**Affiliations:** 1 Department of Research and Innovation, MATIS, Reykjavík, Iceland; 2 University of Technology of Compiègne, Compiègne, France; 3 Faculty of Food Science and Nutrition, University of Iceland, Reykjavík, Iceland; 4 The Agricultural University of Iceland, Hvanneyri, Iceland

**Keywords:** air, *Flavobacterium*, genome, Iceland, volcanic islands Surstey

## Abstract

A novel bacterium, designated 19SA41, was isolated from the air of the Icelandic volcanic island Surtsey. Cells of strain 19SA41 are Gram-stain-negative, strictly aerobic, non-motile rods and form pale yellow-pigmented colonies. The strain grows at 4–30 °C (optimum, 22 °C), at pH 6–10 (optimum, pH 7.5) and with 0–4% NaCl (optimum, 0.5%). Phylogenetic analyses based on 16S rRNA gene sequences showed that 19SA41 belonged to the genus *Flavobacterium* and is most similar to *Flavobacterium xinjiangense* DSM 19743^T^, with a sequence similarity of 96.52%. The new strain contained iso-C_15 : 0_ (22%) and summed feature 3 (C_16∶1_ω6c/C_16∶1_ω7c) (20%) as the predominant fatty acids. The major respiratory quinone was menaquinone-6 (100%). The polar lipid profile consisted of phosphatidylethanolamine and several uncharacterized amino lipids, glycolipids and lipids. The genome of the new strain was 4.01 Mbp, and its G+C content was 33.2 mol%. Based on characterization and comparative results, using a polyphasic taxonomic approach, we propose that the new isolate represents a novel species of the genus *Flavobacterium* with the name *Flavobacterium aerium* sp. nov. The type strain is ISCaR-07695^T^ (=DSM 116640^T^ =UBOOC-M-3567^T^).

## Introduction

The genus *Flavobacterium* was first established by Bergeh *et al*. [1] in the family *Flavobacteriaceae* of the phylum Bacteroidota. This genus comprises 312 species with validly published and correct names [2] (accessed in September 2024).

The properties of this genus have been amended several times and broadly include Gram-stain-negative, rod-shaped, yellow to orange-coloured colonies, aerobic and non-sporulating bacteria [3,6]. The species of this genus are characterized by the presence of menaquinone-6 (MK6) as the predominant respiratory quinone; phosphatidylethanolamine (PE) as the principal polar lipid; summed feature 3 (C_16∶1_ω6c/C_16∶1_ω7c), anteiso-C_15 : 0_ and iso-C_15 : 0_ as the main fatty acids and DNA G+C content in the range of 32.0–37.0 mol% [4,7]. *Flavobacteria* are a ubiquitous and metabolically diverse group of bacteria that are found in a wide range of environments, including freshwater, marine and soil habitats. Most *Flavobacterium* are harmless, but some are opportunistic or true pathogens and cause disease in a wide variety of organisms, including fish and humans [8,9].

This study presents the taxonomic characterization of a new bacterial strain 19SA41, isolated from the air above the Icelandic volcanic island Surtsey during a sampling expedition to investigate the diversity of culturable airborne bacteria on the island.

Morphological, physiological, chemotaxonomic and molecular studies revealed that the isolate should be placed in the genus *Flavobacterium*. Systematic studies on 16S rDNA similarity and DNA–DNA reassociation further distinguished the isolate from known species in the genus *Flavobacterium*. The name *Flavobacterium aerium* sp. nov. is proposed for this isolate.

## Method

### Isolation and culture conditions

A novel bacterium, designated 19SA41, was isolated from an air sample collected on Surtsey island in 2019 [10]. This strain was collected from the air above the Tangi station, located on the rock peninsula of the island (11 m elevation, GPS: 63°18.455´N,20°36.011´W). Surtsey is a neo-volcanic island on the South coast of Iceland, which was formed after a 4-year submarine volcanic eruption from 1963 to 1967 [11]. Purification of the 1162 strains recovered from the air samples was achieved by repeated streaking on Reasoner’s 2A (R2A) medium plate [12]. Out of these isolates, the strain described here was selected for further characterization, as it showed low 16S rRNA gene sequence similarity with blast (~96%) to other known isolates in the 16S ribosomal RNA sequences database (https://blast.ncbi.nlm.nih.gov/Blast.cgi). The strain 19SA41 was primarily isolated and maintained on R2A agar medium at 22 °C and stored at −80 °C in R2A broth with 20% (v/v) glycerol. It has been deposited in the Icelandic Strain Collection and Records (ISCaR, http://iscar.matis.is/) under ISCaR-07695. The two reference strains, *Flavobacterium xinjiangense* DSM 19743^T^ (FX) and *Flavobacterium aquatile* DSM 1132^T^ (FA), were obtained from the German Collection of Microorganisms and Cell Cultures GmBh.

### Genomic and phylogenetic analyses

Total genomic DNA was extracted using MasterPure DNA Purification Kit (Epicentre, Madison, WI, USA), following the manufacturer’s instructions. The whole-genome sequence of strain 19SA41 was obtained by Illumina MiSeq sequencing (Illumina Inc., San Diego, CA, USA). Genomic libraries were prepared using the Illumina DNA Prep kit. The output reads were qualified with FastQC [13] and Trimmomatic (version 0.39) [14]. Reads were assembled with SPAdes (version 3.12.0) [15], and their quality was assessed with QUAST (version 5.2.0) [16]. The genome sequence of strain 19SA41 has been deposited in GenBank under the accession number JARQWR000000000.

To determine the phylogenetic placement of strain 19SA41, its 16S rRNA gene sequence was retrieved from the whole-genome sequence with RNAmmer (version 1.2) [17]. This sequence was deposited in the GenBank database (accession number OQ703064) and compared with 16S rRNA sequences obtained from the TrueBac database [18] to identify the closest relative species. To assess genomic similarities and confirm species boundaries, the genomic G+C content of strain 19SA41 and its closest type strains were calculated from their whole-genome sequences. The average nucleotide identity (ANI) values were calculated using the ANI Calculator from EzBioCloud [19]. Digital DNA–DNA hybridization (dDDH) values were determined using the Genome-to-Genome Distance Calculator (version 3.0) online tool from the DSMZ [20]. Prokka version 1.14.6 [21] was used with default settings for genome annotation, while the Rapid Annotations using Subsystems Technology (RAST) and the SEED Viewer [22] were employed to gain a comprehensive understanding of the organism’s functional capabilities.

To further elucidate the evolutionary relationship and taxonomic placement of strain 19SA41 within the genus *Flavobacterium*, a phylogenetic analysis was conducted. Multiple sequence alignment of the 16S rRNA sequences of all available validly published *Flavobacterium* species was done by the muscle [23] method using MEGAX software [24]. Phylogenetic trees based on the neighbour-joining [25], maximum parsimony [26], and maximum likelihood [27] algorithms, with 1000-replicate bootstrap values, were constructed using the megax software. The evolutionary distance was calculated using Kimura’s two-parameter model. *Myroides odoratus* served as an outgroup of the family of *Flavobacteriaceae* [28].

### Morphology

Colony morphology was determined on R2A agar after 24 h of cultivation at 22 °C using a Zoom 2000 stereomicroscope (Leica Microsystems, Wetzlar, Germany). The cellular morphology of all strains was observed by a polarized light microscope (Olympus BX51, Olympus Life Science, Center Valley, PA, USA) at 1000× magnification (ocular CWH10X-H/20 objective 100 x/1.25/Oil Ph3). Gram staining was performed on 24-hour-old cells using the BBL Gram Stain kit (BD), following the manufacturer’s instructions.

### Temperature, NaCl and pH tolerance

The range and optima of growth at different temperatures were determined by visual inspection of the new isolate 19SA41 and the reference strains on R2A agar plates after 1 week of incubation at 4, 7, 12, 17, 22, 30, 35 and 44 °C. The range of pH and NaCl was determined by visual inspection of growth in liquid R2A for the 19SA41 strain after 1 week of incubation at 22 °C for 19SA41. Tested pH ranged from 4 to 12 with 1-unit intervals and NaCl concentrations from 0% to 4% with 0.5% intervals. To test growth in different pH levels, the medium was prepared by adjusting its pH to the desired levels using HCl and NaOH, followed by sterilization through filtration. The growth optima for pH and NaCl were assessed in liquid R2A for the three strains by regular measurements of the optical density at 600 nm using a Novaspec III Spectrophotometer (Biochrom Ltd., Cambridge, UK), while the strains were growing in liquid R2A in flasks under 160 r.p.m. agitation, at 22 °C for 19SA41 and FA and 12 °C for FX. The tested pH for the optima was 7, 7.5 and 8, whereas the tested NaCl concentrations were 0, 0.5 and 1%. All growth analyses were performed in triplicates with non-inoculated media in the same condition, as negative controls.

### Biochemical and physiological characteristics

19SA41, FA and FX were phenotypically characterized by the most relevant tests recommended for the description of new taxa within the family *Flavobacteriaceae*. These tests comprised a comprehensive biochemical characterization of isolates with the testing of enzymatic activity, carbohydrate fermentation, and carbon sources utilization determined using API ZYM, API 20 NE and API 20E strips (bioMérieux), according to the manufacturer’s instructions. Further tests were performed: oxidase activity was assessed using an oxidase reagent kit (Difco BBL), Catalase test was performed by observing the formation of bubbles after the addition of 3% (v/v) H_2_O_2_ to fresh cells. The methyl red test was performed by inoculating fresh cells into MRVP broth and observing the change in colour when adding five drops of methyl red agent after 4 days of incubation. Hydrolysis of casein was checked by inoculating fresh cells on Skim Milk agar and observing the area surrounding the growth zone after 4 days of incubation. The same principle was used in the starch hydrolysis test, observing the area surrounding the growth zone after incubation of fresh cells on Starch agar. The detection of flexirubin-type pigments using 20 % KOH and extracellular glycans using the Congo red absorption test was performed according to McCammon and Bowman [29]. The motility test was performed by inoculating fresh cells into semi-solid Sulfide Indole Motility medium and observing the growth zone after 1 week of incubation. Gliding motility was assessed by microscopic observation of a hanging drop from a fresh TSB culture. In addition, anaerobic growth was tested after 1 week of incubation on R2A agar using a GasPak (BD). All incubations were performed at 22 °C for 19SA41 and FA and 12 °C for FX.

### Antibiotic susceptibility

The susceptibility of 19SA41 to antibiotics was tested in triplicates on R2A agar plates using antibiotic discs (Oxoid) at 5 µg per disc for novobiocin; 15 µg per disc for oleandomycin, lincomycin and erythromycin; 30 µg per disc for gentamycin, kanamycin, rifampicin, cephalothin, tetracycline and vancomycin; and 100 µg per disc for carbenicillin. The interpretation of the results was based on the latest guidelines and recommendations from recent literature.

### Chemical characterization

The analyses of cellular fatty acids (GC-MS), lipoquinones (HPLC) and polar lipids (two-dimensional silica gel thin layer chromatography) composition were carried out on freeze-dried cells from actively growing liquid cultures by the Identification Service of Leibniz-Institute DSMZ – Deutsche Sammlung Von Mikroorganismen und Zellkulturen GmbH, Braunschweig, Germany. Details on the protocols can be found online (https://www.dsmz.de/).

## Results and discussion

### Genomic and phylogenetic analyses

The draft assembly of 19SA41 contains 42 contigs of 4 004 540 bases in total (N50 : 315864), and the nearly full-length 16S rRNA gene sequence of 1502 bp was retrieved from this whole-genome sequence. Alignment of the 16S rRNA gene sequence revealed that strain 19SA41 shares similarities with *Flavobacterium xinjiangense* (96.38%), followed by *Flavobacterium urumqiense* (96.31%), *Flavobacterium kingsejongi* (96.10%), *Flavobacterium rhamnosiphilum* (95.88%) and *Flavobacterium sinopsychrotolerans* (95.79%). Additionally, strain 19SA41 shows 95.33% sequence identity with *Flavobacterium aquatile*, the type species of the genus. In this study, comparative tests were performed with the most closely related strain, *Flavobacterium xinjiangense*, and the type species of the genus, *Flavobacterium aquatile*.

The genome size of strain 19SA41 is comparable to those of *Flavobacterium aquatile* DSM 1132^T^ (3.49 Mb) and *Flavobacterium xinjiangense* DSM 19743^T^ (3.9 Mb) (Table 1), indicating a similar genomic structure. The G+C content of 19SA41 (33.18%) was within the range of *Flavobacterium aquatile* DSM 1132^T^ (32.23%) and *Flavobacterium xinjiangense* DSM 19743^T^ (34%) (Table 1) and is consistent with the expected range for the genus *Flavobacterium*. ANI values between strain 19SA41 and *Flavobacterium aquatile* DSM 1132^T^ and *Flavobacterium xinjiangense* DSM 19743^T^ were 72.96 and 12.47%, respectively (Table 1). These values are significantly below the 95–96% threshold typically used for species delimitation [30,32], suggesting that strain 19SA41 represents a distinct species within the genus. This conclusion is further supported by dDDH values, which were 18.8 and 19.9% for comparisons with *Flavobacterium aquatile* DSM 1132^T^ and *Flavobacterium xinjiangense* DSM 19743^T^, respectively (Table 1). These values are well below the 70% threshold for species definition, reinforcing the novel species status of strain 19SA41 within the genus *Flavobacterium*. Additionally, most functional RNA categories remain similar across the tested strains, and strain 19SA41 contains two repeat regions, whereas FA and FX have none (Table 1). These repeat regions contribute to gene regulation and may confer unique genomic features or adaptive advantages not present in strains FA and FX.

**Table 1.** Phylogenetic and genomic characteristics that differentiate strain 19SA41 from FA and FX

Characteristics	19SA41	FA	FX
Genome size (Mbp)	4.01	3.49	3.90
G+C content (mol%)	33.18	32.23	34.00
ANI to strain 19SA41 (%)	100	72.96	72.47
dDDH (generalized linear model-based) to isolate 19SA41 (%)	100	18.8	19.9
16S rRNA gene similarity (%)	100	95.33	96.38
Coding sequences (CDS)	3555	3422	3173
Repeat region	2	0	0
Ribosomal RNA (rRNA)	3	4	5
Transfer RNA (tRNA)	43	45	38
Transfer-messenger RNA (tmRNA)	1	1	1

Based on the RAST analysis and the SEED viewer results, the main subsystem features are very similar among strains 19SA41, FA and FX (Table S1). However, some differential features could be noticed (Table 2), revealing advantages in nutrient utilization and environmental adaptability. Strain 19SA41 exhibits a higher number of functional genes associated with arginine metabolism, which can serve as important sources of carbon and nitrogen, facilitating bacterial adaptation to acidic environments [33]. Additionally, this strain possesses enhanced biotin synthesis capabilities, providing metabolic versatility that increases its survival advantage in environments with limited nutrient availability [34]. Furthermore, its ability to efficiently scavenge iron, supported by siderophore production, enhances survival in iron-limited conditions. The presence of detoxification genes also contributes to its resilience against environmental stressors, positioning strain 19SA41 as a highly adaptable and competitive organism in varying ecological niches compared to strains FA and FX. These adaptations enable strain 19SA41 to thrive in the stressful, low-nutrient air environment from which it was sampled. Moreover, it holds potential biotechnological applications, such as the use of siderophores in medicine and industry [35].

**Table 2.** Differential features of metabolic subsystems among strains 19SA41, FA and FX. Counts represent the number of functional genes identified in each category

		Counts		
Subsystem feature	Subcategory	19SA41	FX	FA
Amino acids and derivatives	Arginine, urea cycle and polyamines	22	6	10
Respiration	Biotin	9	0	0
Stress response	Detoxification	5	1	1
Iron acquisition and metabolism	Iron acquisition and metabolism – no subcategory	9	0	0
	Siderophores	1	0	0

**Fig. 1.**
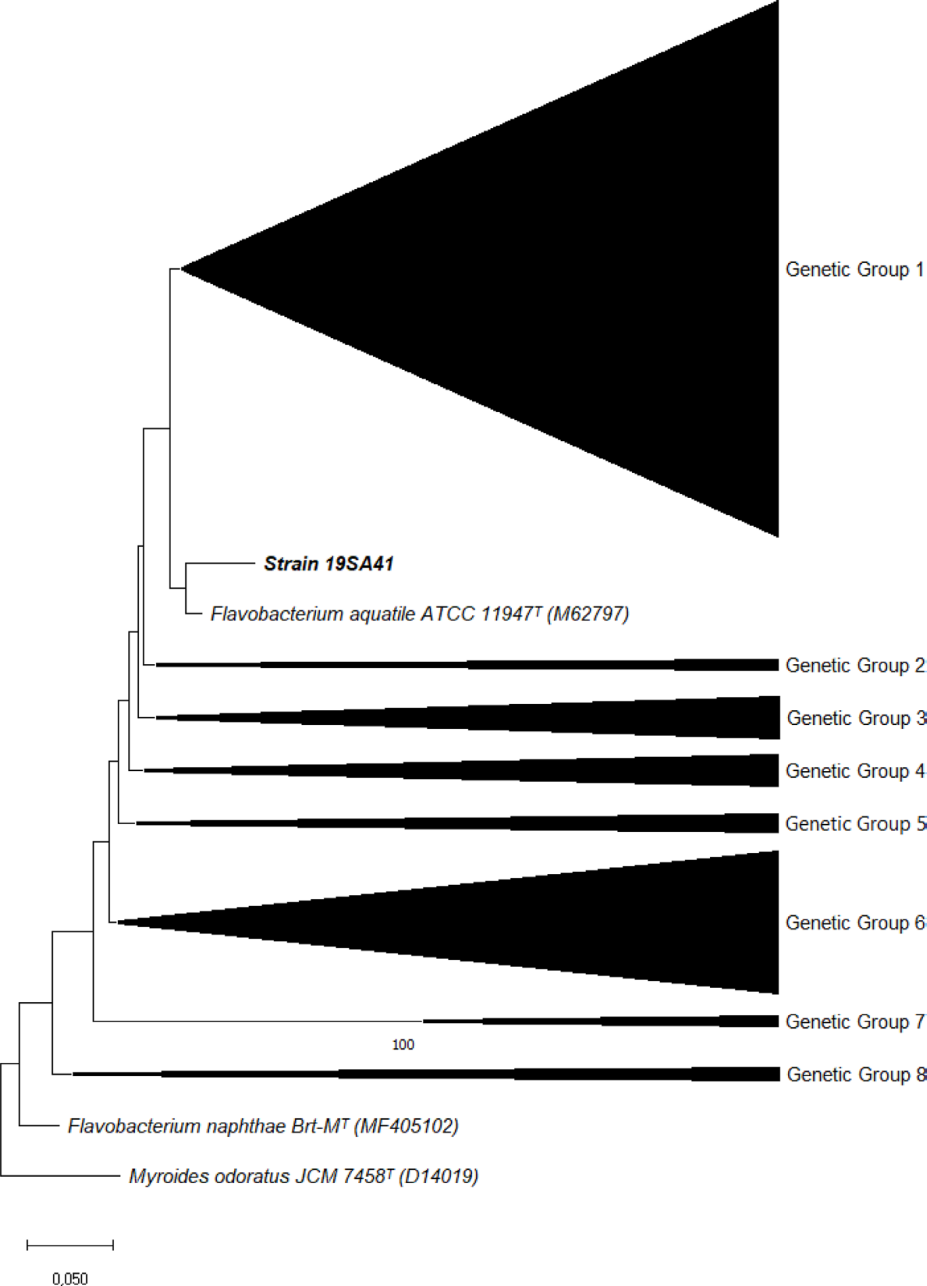
Maximum-likelihood phylogenetic tree based on 16S rRNA gene sequences showing the position of strain 19SA41 and related taxa. Bootstrap values >70% based on 1000 replicates are shown at branching points. Filled circles indicate nodes also recovered using neighbour-joining and maximum-parsimony algorithms. Bar, 0.020 substitutions per nucleotide position. ^T^: type strain. The full phylogenetic tree can be found in the Supplementary Material.

The phylogenetic tree based on 16S rRNA gene sequences (Fig. 1) places strain 19SA41 within the genus *Flavobacterium*, but it does not cluster closely with one of its highest sequence identity matches, *Flavobacterium xinjiangense*. This positioning reflects the evolutionary divergence within the genus. The tree, along with ANI and dDDH results, supports the conclusion that strain 19SA41 represents a novel species within the genus.

### Morphology

The colonies of strain 19SA41 were pale yellow, circular and raised, with entire margins and a diameter of ~1.5 mm. The colour of the colonies of 19SA41 distinguishes them from *Flavobacterium aquatile* DSM 1132^T^ and *Flavobacterium xinjiangense* DSM 19743^T^ with a much lighter yellow. The tested strains were Gram-stain-negative, and their cells were rod-shaped, measuring ~1 µm wide and 2 µm long. Phenotypic characteristics that distinguish strain 19SA41 from the reference strains FA and FX are presented in Table 3.

**Table 3.** Results summary of the tests that distinguish 19SA41 from FA and FX. +, positive; −, negative

	19SA41	FA	FX
**Morphology and growth parameters**			
Colonies	Pale yellow	Yellow	Yellow
Temperature range (optimum)	4–30 °C (22 °C)	4–22 °C (12 °C)	4–32.5 °C (22 °C)
pH optimum	7.5	7.5	7
NaCl optimum (%)	0.50	0.50	1
**Chemicalcharacterization**			
Polar lipids	PE, AL, GL and L	PE, APL, AL, PL and L	na
Cellular fatty acids (> 8%)	Iso-C_15 : 0_ and summed feature 3	Summed feature 3	Iso-C15 : 0, iso-C17 : 0 3-OH and iso-C15 : 1 G (*Zhu et al.*, 2003)
Menaquinones	MK6 (100%)	MK6 (100%)	na
**Enzyme production**			
*β*-Galactosidase	+	–	–
*α*-Glucuronidase	–	+	+
*β*-Glucosidase	–	+	+
*N*-Acetyl-*β*-glucosaminidase	–	+	+
*α*-Fucosidase	+	–	–
Gelatinase	+	–	–
Voges–Proskauer	–	+	+
**Carbon source assimilation**			
Arabinose	–	+	+
Mannitol	–	+	+
Potassium gluconate	–	+	+
Capric acid	–	+	+
Adipic acid	–	+	+
Phenylacetic acid	–	+	+

### Temperature, NaCl and pH tolerance

Strain 19SA41 grows at tested temperatures ranging from 4 to 30 °C, with an optimum growth temperature of 22 °C. The growth occurred at a pH ranging from 6 to 10, with an optimum of 7.5, and at an NaCl concentration from 0 to 4%, with an optimum of 0.5% (Fig. S1A and B). Under optimal conditions, strain 19SA41 has a maximal growth rate of 0.4 h^−1^ and a generation time of 103 min (Fig. S1C).

### Biochemical and physiological characteristics

Strain 19SA41 distinguishes itself from the reference strains by producing the enzymes *β*-galactosidase and *α*-fucosidase but not producing the enzymes *α*-glucuronidase, *β*-glucosidase and *N*-acetyl-*β*-glucosaminidase. Additionally, it showed a negative Voges–Proskauer test result and does not assimilate arabinose, mannitol, potassium gluconate, capric acid, adipic acid and phenylacetic acid. The test results that distinguish 19SA41 from FA and FX can be found in Table 3. In addition, all test results are available in Tables S2–5.

### Antibiotic susceptibility

Strain 19SA41 and its closest reference strains were resistant to all tested antimicrobials except tetracycline 30 µg. Results can be found in Table S6.

### Chemical characterization

The predominant cellular fatty acids of strain 19SA41 were iso-C_15 : 0_ (22%) and summed feature 3 (C_16∶1_ω6c/C_16∶1_ω7c) (20%). The fatty acid composition of strain 19SA41 and its reference strains is shown in Table 3 and Fig. S2. The fatty acid profile of strain 19SA41 differed markedly from those of the two reference strains by higher amounts of iso-C_15 : 0_3-OH, iso-C_16 :  0_, C_16  :  0_ 3-OH and summed feature 9 (iso-C_17∶1_ω9c/C_16∶0_ 10 methyl), and the absence of anteiso-C_17  : 1_ ω9c, iso-C_17  : 1_ ω9c and summed feature 8 (C_18∶1_ω7c/C_18∶1_ω6c). Strain 19SA41 exhibited a complex polar lipid profile consisting of PE, eight uncharacterized aminolipids (AL), six uncharacterized glycolipid (GL) and five uncharacterized lipids (L) Fig. S3. The polar lipid profile of strain 19SA41 differed from those of *Flavobacterium xinjiangense* DSM 19743^T^ by the presence of glycolipids and the absence of aminophospholipid and phospholipid (Table 3). The respiratory quinones detected in strain 19SA41 were MK6 (100%).

## Conclusion

To conclude, strain 19SA41 had phenotypic characteristics that differentiated it from the other species of *Flavobacterium*, such as the colour of its colonies, the production of *α*-fucosidase and the negative Voges–Proskauer test. Considering the size of its genome, the dDDH and ANI values, the low 16S RNA gene sequence similarity to the closest relative species and based on the differences in the composition of polar lipids and cellular fatty acids, strain 19SA41 represents a novel species of the genus *Flavobacterium*, for which the name *Flavobacterium aerium* sp. nov. is proposed.

### Description of *Flavobacterium aerium* sp. nov.

*Flavobacterium aerium* (a.e’ri.um. L. neut. adj. aerium, belonging to the air)

Gram-negative rods, 2–3 µm in length and 0.5–1 µm in width. Aerobic, non-flagellated and gliding. Colonies on solid Reasoner’s 2A (R2A) medium after incubations at 22 °C are pale yellow, 1.5 mm wide and circular with entire margins. Growth occurs at 4–30 °C (optimum, 22 °C), pH 6–10 (optimum, 7.5) and 0–4% NaCl (w/v; optimum, 0.5%) on R2A medium, with a generation time of 103 min under optimal conditions.

Flexirubin pigments are not detected. The following biochemical tests are negative: Congo red absorption, methyl-red test, production of hydrogen sulfide and Voges–Proskauer test. Does not produce these enzymes: arginine dihydrolase, lysine decarboxylase, lipase, trypsine, *α*-chymotrypsine, *α*-galactosidase, *α*-glucuronidase, *β*-glucuronidase, *N*-acetyl-*β*-glucosaminidase, *α*-mannosidase, *β*-glucosidase, ornithine decarboxylase, urease, tryptophanase and amylase. Does not ferment glucose, mannose, inositol, sorbitol, rhamnose, sucrose, melibiose, amygdaline and arabinose. Does not reduce nitrates to nitrites and nitrates to nitrogen, does not assimilate arabinose, mannitol, potassium gluconate, capric acid, adipic acid and phenylacetic acid.

Produce the enzymes: tryptophan-deaminase, gelatinase, phosphatase alkaline, esterase, esterase lipase, leucine arylamidase, valine arylamidase, caseinase, catalase, oxidase, cystine arylamidase, acid phosphatase, naphtol-AS-BI-phosphohydrolase, *β*-galactosidase and *α*-fucosidase. Assimilates citrate, glucose, *N*-acetyl-glucosamine, maltose, mannose, malic acid and trisodium citrate.

The main fatty acids are iso-C_15 : 0_ and summed feature 3 (C_16∶1_ω6c/C_16∶1_ω7c). The polar lipids are PE, AL, glycolipid and lipid. The respiratory quinones are MK6 (100%). The type strain 19SA41 was isolated from the air of Surtsey island, a neo-volcanic-island on the south coast of Iceland. The G+C content of the DNA of the type strain is 33.18%.

The type strain 19SA41, deposited as ISCaR-07695^T^, DSM 116640^T^ and UBOOC-M-3567^T^, has the genome accession number JARQWR000000000 and the 16S rRNA gene sequence accession number OQ703064.

## supplementary material

10.1099/ijsem.0.006647 Uncited Supplementary Material 1.

## Abbreviations

AL: aminolipids
ANI: average nucleotide identity
dDDH: Digital DNA–DNA hybridization
MK6: menaquinone-6
PE: phosphatidylethanolamine
R2A: Reasoner’s 2A
RAST: Rapid Annotations using Subsystems Technology
