# Supplementary material for: Flavobacterium aerium sp. nov., a bacterium isolated from the air of the Icelandic volcanic island Surtsey
Source: Int J Syst Evol Microbiol. 2025 Jan 23;75(1):006647. doi: 10.1099/ijsem.0.006647 (PMC11756700; doi:10.1099/ijsem.0.006647)
Supplement: Uncited Supplementary Material 1. [file ijsem-75-06647-s001.pdf]

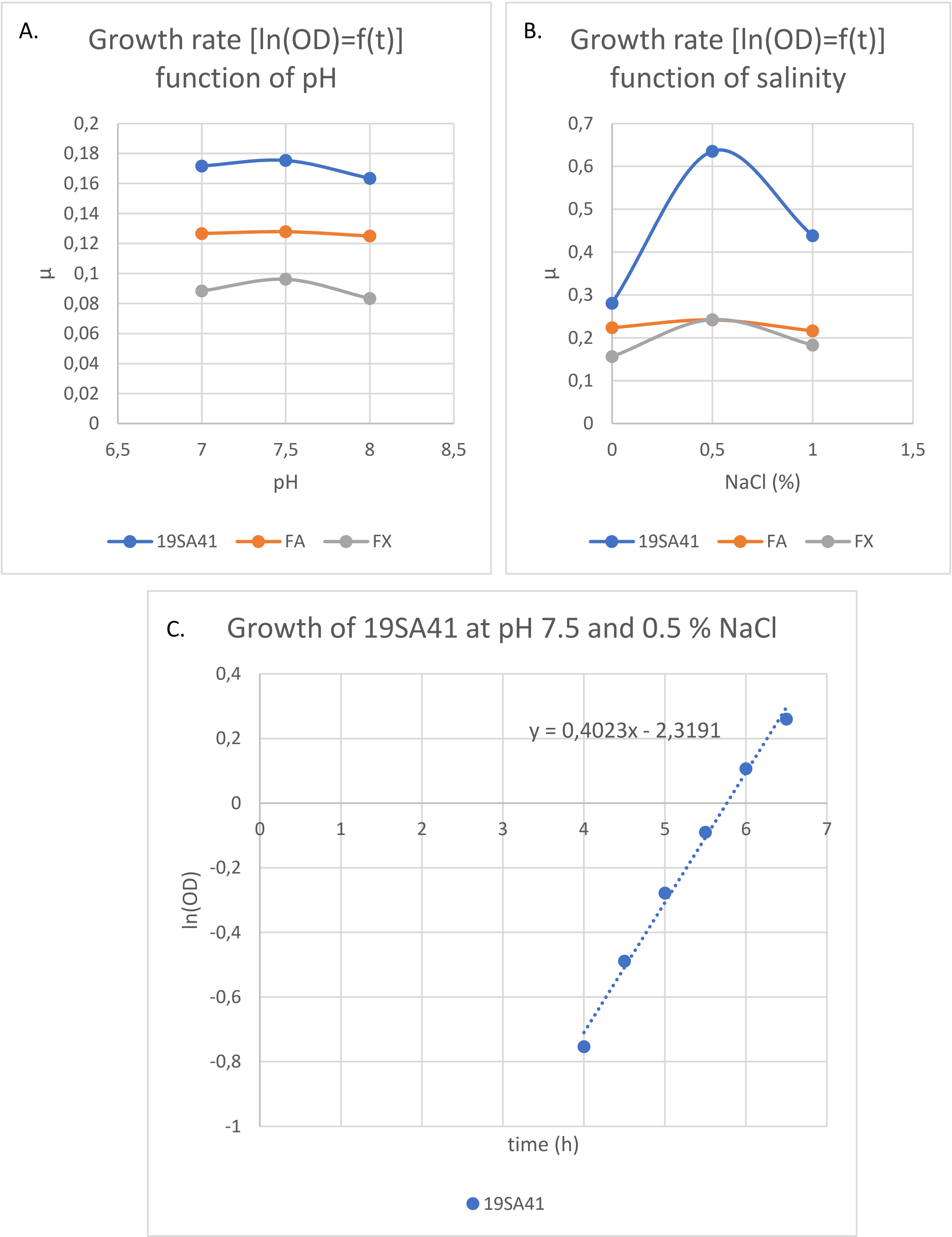

Figure 1: Growth rates of the strain 19SA41 function of pH (A) and Salinity (B); and growth of the strain 19SA41 in optimal conditions at pH 7.5 and with 0.5 % NaCl (C). FA: *Flavobacterium aquatile* DSM 1132^{T}; FX: *Flavobacterium xinjiangense* DSM 19743^{T}.

A.

RT	Response	Ar/Ht	RFact	ECL	Peak Name	Percent	Comment1	Comment2	GC-MS
1.658	4.793E+8	0.026	----	7.019	SOLVENT PEAK	----	< min rt		
2.214	221	0.021	----	8.180		----	< min rt		
2.607	175	0.025	1.245	9.000	9:0	0.05	ECL deviates 0.000	Reference	Not confirmed
3.006	586	0.026	----	9.834		----			
4.226	3962	0.029	----	11.538	unknown 11.543	----	ECL deviates -0.005		
4.288	415	0.032	1.059	11.609	12:0 iso	0.09	ECL deviates 0.000	Reference -0.002	OK
4.632	1034	0.042	1.040	11.998	12:0	0.22	ECL deviates -0.002	Reference -0.004	OK
5.246	2954	0.032	----	12.550		----			
5.317	981	0.035	1.013	12.614	13:0 iso	0.21	ECL deviates 0.000	Reference -0.003	OK
5.671	1850	0.033	1.001	12.932	13:1 at 12-13	0.39	ECL deviates -0.004		Not confirmed
6.339	564	0.040	----	13.444		----			
6.495	8892	0.036	----	13.561	unknown 13.565	----	ECL deviates -0.004		
6.572	6009	0.037	0.978	13.619	14:0 iso	1.23	ECL deviates 0.000	Reference -0.003	OK
7.081	1472	0.036	0.968	14.000	14:0	0.30	ECL deviates 0.000	Reference -0.003	OK
7.746	34461	0.040	0.958	14.441	15:1 iso G	6.90	ECL deviates 0.001		Identified as 15:1 ISO w10c
7.877	1176	0.040	0.956	14.527	15:1 anteiso A	0.23	ECL deviates 0.000		Identified as 15:1 ANTEISO w10c
8.025	109549	0.040	0.954	14.625	15:0 iso	21.85	ECL deviates 0.002	Reference -0.001	OK
8.159	9797	0.038	0.952	14.714	15:0 anteiso	1.95	ECL deviates 0.001	Reference -0.003	OK
8.372	4541	0.052	0.950	14.855	15:1 w6c	0.90	ECL deviates -0.001		OK
8.594	7415	0.039	----	15.002	15:0	----	ECL deviates 0.002		OK
8.788	3912	0.054	0.945	15.120	14:0 iso 3OH	0.77	ECL deviates 0.001		OK
9.343	12417	0.051	0.941	15.458	16:1 iso H	2.44	ECL deviates -0.003		Identified as 16:1 ISO w6c
9.550	1297	0.040	----	15.584		----			
9.620	24963	0.041	0.938	15.627	16:0 iso	4.90	ECL deviates 0.000	Reference -0.003	OK
9.977	103929	0.060	0.936	15.844	Sum In Feature 3	20.34	ECL deviates -0.008	16:1 w6c/16:1 w7c	Double peak: Identified as 16:1 w7c and 15:1 ISO 2OH
10.132	5376	0.060	----	15.939		----			
10.233	7186	0.042	0.935	16.000	16:0	1.40	ECL deviates 0.000	Reference -0.003	OK
10.460	48368	0.043	0.934	16.132	15:0 iso 3OH	9.44	ECL deviates -0.002		OK
10.608	2539	0.044	0.933	16.219	15:0 2OH	0.50	ECL deviates 0.000		OK
10.944	16331	0.043	0.932	16.415	Sum In Feature 9	3.18	ECL deviates -0.001	17:1 iso w9c	Identified as 17:1 ISO w7c
11.071	6940	0.055	0.931	16.490	Sum In Feature 4	1.35	ECL deviates 0.004	17:1 anteiso	Identified as 17:1 ISO w5c
11.228	4276	0.049	----	16.581	unknown 16.582	----	ECL deviates -0.001		
11.311	1146	0.040	0.930	16.630	17:0 iso	0.22	ECL deviates 0.000	Reference -0.003	OK
11.468	332	0.032	0.930	16.722	17:0 anteiso	0.06	ECL deviates -0.001	Reference -0.004	OK
11.597	1467	0.044	0.930	16.797	17:1 w8c	0.29	ECL deviates 0.005		OK
11.706	13582	0.045	0.929	16.860	17:1 w6c	2.64	ECL deviates 0.000		OK
12.199	23728	0.047	0.928	17.146	16:0 iso 3OH	4.61	ECL deviates -0.004		OK
12.847	11368	0.046	0.928	17.516	16:0 3OH	2.21	ECL deviates -0.003		OK
13.286	8498	0.079	0.928	17.767	18:1 w9c	1.65	ECL deviates -0.002		OK
13.455	1692	0.050	0.928	17.864	Sum In Feature 8	0.33	ECL deviates 0.006	18:1 w6c	Identified as 18:1 w7c
13.543	719	0.042	0.928	17.914	18:1 w5c	0.14	ECL deviates -0.005		OK
13.694	1038	0.053	0.928	18.001	18:0	0.20	ECL deviates 0.001	Reference	OK
13.968	43768	0.047	0.929	18.158	17:0 iso 3OH	8.50	ECL deviates -0.003		OK
14.136	1908	0.047	0.929	18.254	17:0 2OH	0.37	ECL deviates 0.000		Not confirmed
14.616	772	0.038	0.930	18.530	17:0 3OH	0.15	ECL deviates -0.006		OK
----	103929	---	----	----	Summed Feature 3	20.34	16:1 w7c/16:1 w6c	16:1 w6c/16:1	
----	6940	---	----	----	Summed Feature 4	1.35	17:1 iso I/anteiso B	17:1 anteiso	
----	1692	---	----	----	Summed Feature 8	0.33	18:1 w7c	18:1 w6c	
----	16331	---	----	----	Summed Feature 9	3.18	17:1 iso w9c	16:0 10-methyl	

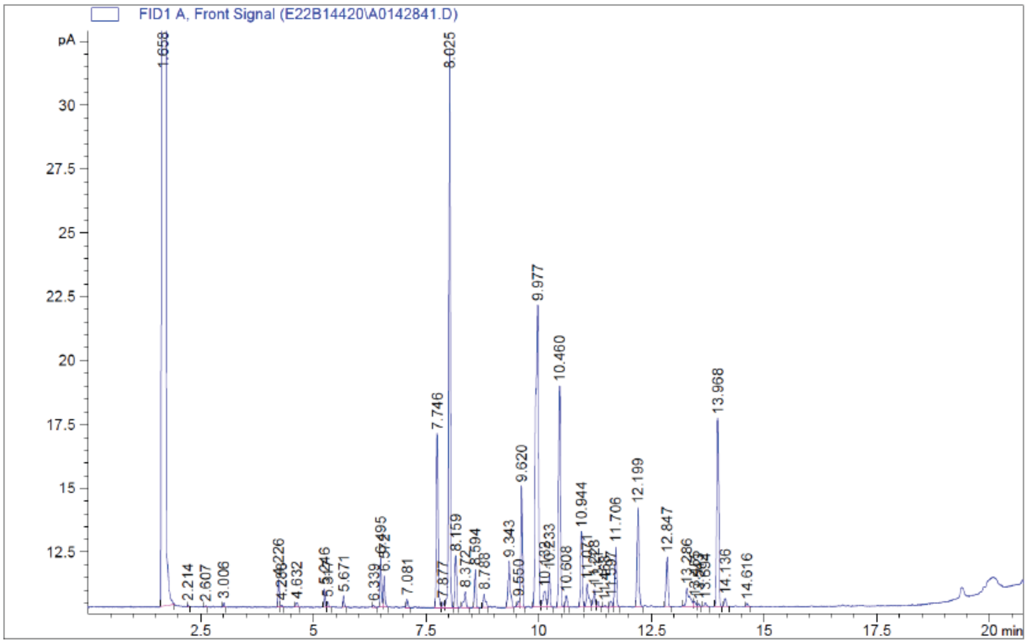

B.

RT	Response	Ar/Ht	RFact	ECL	Peak Name	Percent	Comment1	Comment2	GC-MS
1.657	4.79E+8	0.027	----	7.019	SOLVENT PEAK	----	< min rt		
2.213	238	0.022	----	8.179		----	< min rt		
2.606	179	0.021	1.246	8.997	9:0	0.07	ECL deviates -0.003	Reference -0.001	Not confirmed
3.006	476	0.024	----	9.832		----			
3.695	200	0.024	1.099	10.916	Sum In Feature 2	0.07	ECL deviates 0.002	12:0 aldehyde ?	Not confirmed
4.225	1695	0.028	----	11.536	unknown 11.543	----	ECL deviates -0.007		
4.302	375	0.028	1.059	11.623	12:0 iso	0.13	ECL deviates 0.014	Reference 0.014	Not confirmed
4.632	2130	0.040	1.040	11.996	12:0	0.70	ECL deviates -0.004	Reference -0.004	OK
5.245	5897	0.033	----	12.547		----			
5.318	575	0.031	1.013	12.612	13:0 iso	0.18	ECL deviates -0.002	Reference -0.002	OK
5.671	1633	0.031	1.001	12.929	13:1 at 12-13	0.52	ECL deviates -0.007		Not confirmed
6.334	651	0.032	----	13.438		----			
6.495	4380	0.035	----	13.559	unknown 13.565	----	ECL deviates -0.006		
6.572	8025	0.038	0.979	13.617	14:0 iso	2.48	ECL deviates -0.002	Reference -0.003	OK
6.830	702	0.037	----	13.810		----			
7.000	250	0.034	----	13.938		----			
7.081	1842	0.035	0.969	13.999	14:0	0.56	ECL deviates -0.001	Reference -0.002	OK
7.745	9938	0.041	0.959	14.438	15:1 iso G	3.01	ECL deviates -0.002		Identified as 15:1 ISO w10c
7.877	3940	0.038	0.957	14.526	15:1 anteiso A	1.19	ECL deviates -0.001		Identified as 15:1 ANTEISO w10c
8.022	23212	0.041	0.956	14.622	15:0 iso	7.00	ECL deviates -0.001	Reference -0.002	OK
8.159	32521	0.039	0.954	14.712	15:0 anteiso	9.80	ECL deviates -0.001	Reference -0.002	OK
8.310	1761	0.036	----	14.813		----			
8.374	16973	0.040	0.951	14.855	15:1 w6c	5.10	ECL deviates -0.001		OK
8.593	11988	0.039	----	15.000	15:0	----	ECL deviates 0.000		OK
8.786	1307	0.045	0.947	15.118	14:0 iso 3OH	0.39	ECL deviates -0.001		OK
9.021	881	0.044	----	15.262		----			
9.343	13600	0.047	0.942	15.457	16:1 iso H	4.05	ECL deviates -0.004		Identified as 16:1 ISO w6c
9.550	1546	0.043	----	15.584		----			
9.619	12824	0.042	0.940	15.626	16:0 iso	3.81	ECL deviates -0.001	Reference -0.003	OK
9.933	84185	0.043	0.938	15.817	Sum In Feature 3	24.95	ECL deviates -0.005	16:1 w7c/16:1 w6c	Identified as 16:1 w7c
10.081	2663	0.069	0.938	15.908	16:1 w5c	0.79	ECL deviates -0.001		OK
10.232	3770	0.043	0.937	16.000	16:0	1.12	ECL deviates 0.000	Reference -0.003	OK
10.458	10942	0.047	0.936	16.132	15:0 iso 3OH	3.23	ECL deviates -0.002		OK
10.612	2046	0.045	0.935	16.223	15:0 2OH	0.60	ECL deviates 0.004		Identified as 15:0 anteiso 3OH
10.943	8232	0.042	0.934	16.416	Sum In Feature 9	2.43	ECL deviates 0.000	17:1 iso w9c	Identified as 17:1 ISO w7c
11.125	3917	0.066	0.933	16.523	17:1 anteiso w9c	1.15	ECL deviates -0.001		Identified as 17:1 ANTEISO w7c
11.232	1121	0.052	----	16.585	unknown 16.582	----	ECL deviates 0.003		
11.589	3788	0.044	0.932	16.794	17:1 w8c	1.11	ECL deviates 0.002		OK
11.708	31692	0.044	0.931	16.864	17:1 w6c	9.32	ECL deviates 0.004		OK
12.199	22648	0.046	0.930	17.148	16:0 iso 3OH	6.65	ECL deviates -0.002		OK
12.846	5533	0.045	0.930	17.518	16:0 3OH	1.62	ECL deviates -0.001		OK
13.285	6457	0.051	0.929	17.770	18:1 w9c	1.89	ECL deviates 0.001		OK
13.379	2639	0.054	0.929	17.824	Sum In Feature 8	0.77	ECL deviates 0.001	18:1 w7c	Identified as 18:1 w7c
13.451	2392	0.063	0.929	17.865	Sum In Feature 8	0.70	ECL deviates 0.007	18:1 w6c	Identified as 18:1 w6c
13.690	1190	0.050	0.929	18.002	18:0	0.35	ECL deviates 0.002	Reference -0.003	OK
13.966	11034	0.047	0.929	18.160	17:0 iso 3OH	3.24	ECL deviates -0.001		OK
14.132	2018	0.043	0.930	18.256	17:0 2OH	0.59	ECL deviates 0.002		Identified as 17:0 anteiso 3OH
14.362	803	0.042	0.930	18.389	18:0 10-methyl, TBSA	0.24	ECL deviates -0.003		Not confirmed
14.615	586	0.048	0.930	18.534	17:0 3OH	0.17	ECL deviates -0.002		OK
----	200	---	----	----	Summed Feature 2	0.07	12:0 aldehyde ?	unknown 10.928	
----	-----	---	----	----		----	16:1 iso I/14:0 3OH	14:0 3OH/16:1 iso I	
----	84185	---	----	----	Summed Feature 3	24.95	16:1 w7c/16:1 w6c	16:1 w6c/16:1 w7c	
----	5031	---	----	----	Summed Feature 8	1.48	18:1 w7c	18:1 w6c	
----	8232	---	----	----	Summed Feature 9	2.43	17:1 iso w9c	16:0 10-methyl	

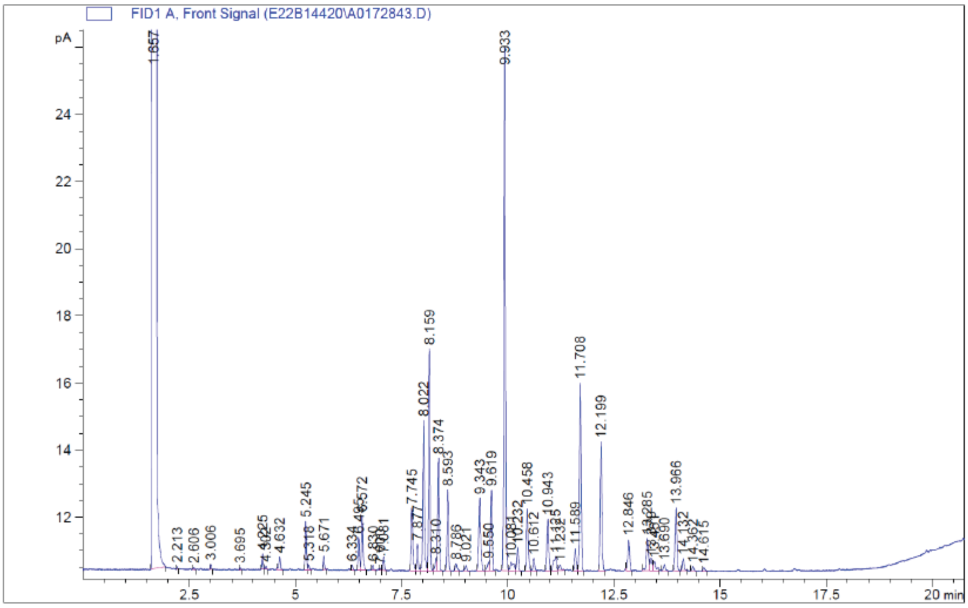

Figure 2. Fatty acids analyses of 19SA41 (A) and *Flavobacterium xinjiangense* DSM 19743^{T} (B)

A.

PE = Phosphatidylethanolamine

AL = Aminolipid

GL = Glycolipid

L = Lipid

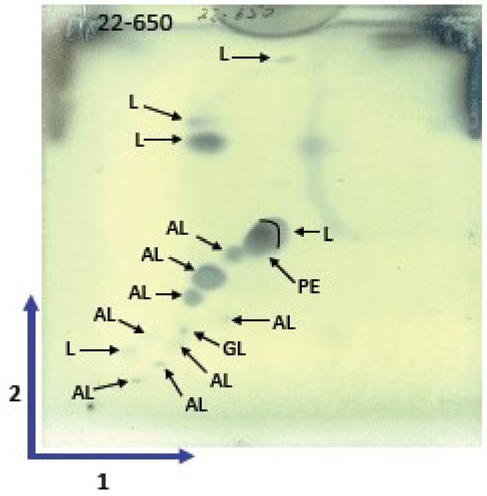

B.

PE = Phosphatidylethanolamine

APL = Aminophospholipid

AL = Aminolipid

PL = Phospholipid

L = Lipid

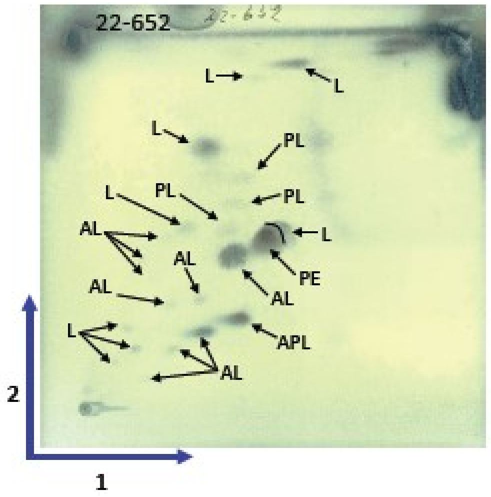

Figure 3. Polar lipid profiles of strain 19SA41 (A) and *Flavobacterium xinjiangense* DSM 19743^{T} (B)

Supplementary Material – Tables

Supplementary Material – Table 1. Subsystem features of strains 19SA41, FA, and FX. Counts represent the number of functional genes identified in each category.

	Counts		
Subsystem Feature	19SA41	FX	FA
Amino Acids and Derivatives	195	177	162
Cofactors, Vitamins, Prosthetic Groups, Pigments	133	153	114
Protein Metabolism	107	140	106
Carbohydrates	67	91	86
Nucleosides and Nucleotides	60	60	58
DNA Metabolism	59	74	50
Respiration	59	55	53
Membrane Transport	41	33	39
RNA Metabolism	35	39	34
Fatty Acids, Lipids, and Isoprenoids	33	32	45
Virulence, Disease and Defense	31	29	28
Cell Wall and Capsule	24	33	24
Phages, Prophages, Transposable elements, Plasmids	24	23	1
Stress Response	17	24	24
Miscellaneous	13	18	15
Phosphorus Metabolism	12	14	12
Metabolism of Aromatic Compounds	11	10	14
Iron acquisition and metabolism	10	0	0
Sulfur Metabolism	9	8	6
Potassium metabolism	8	6	8
Secondary Metabolism	7	6	6
Nitrogen Metabolism	3	31	27
Regulation and Cell signaling	3	4	6
Dormancy and Sporulation	1	1	1
Cell Division and Cell Cycle	0	0	0
Motility and Chemotaxis	0	0	0
Photosynthesis	0	0	0

Supplementary Material – Table 2. Phenotypic characteristics of strain 19SA41 and the reference strains. +, positive; -, negative. Highlighted are the results that distinguish 19SA41 from *F. aquatile* DSM 1132^T and *F. xinjiangense* DSM 19743^T.

Test	Isolate 19SA41	*F. aquatile* DSM 1132^T	*F. xinjiangense* DSM 19743^T
**Pigmentation**	**Pale yellow**	**Yellow**	**Yellow**
Rod size (width x length; µm)	1 x 2	1 x 2	1 x 2
Gram staining	-	-	-
**Temperature range / optimum (°C)**	**4-30/22**	**4-32.5/22**	**4-22/12**
**pH range / optimum**	**6-10/7.5**	**NA/7**	**NA/7.5**
**[NaCl] range / optimum (w/v)**	**0-4/0.5**	**NA/1**	**NA/0.5**
Aerobic growth	-	-	-
Motility	-	-	-
Catalase production	+	+	+
Oxydase production	+	+	+
Flexirubin-type pigment production	-	-	-
Congo red absorption	-	-	-
Methyl-red test	-	-	-
Hydrolysis of gelatin	+	+	+
Hydrolysis of casein	+	+	+
Hydrolysis of starch	-	-	-
Gliding Motility	+	+	+

Supplementary Material – Table 3. Results of the API ZYM strip tests. +, positive; -, negative. Highlighted are the results that distinguish 19SA41 from *F. aquatile* DSM 1132^T and *F. xinjiangense* DSM 19743^T.

Test – Enzyme production	Isolate 19SA41	*F. aquatile* DSM 1132^T	*F. xinjiangense* DSM 19743^T
Control	-	-	-
Phosphatase alkaline	+	+	+
Estérase	+	+	+
Estérase Lipase	+	+	+
Lipase	-	-	-
Leucine arylamidase	+	+	+
Valine arylamidase	+	+	+
Cystine arylamidase	+	+	+
Trypsine	-	-	-
alpha-chymotrypsine	-	-	-
Acid phosphatase	+	+	+
Naphtol-AS-BI-phosphohydrolase	+	+	+
alpha-galactosidase	-	-	-
**beta-galactosidase**	**+**	**-**	**-**
beta-glucuronidase	-	-	-
**alpha-glucuronidase**	**-**	**+**	**+**
**beta-glucosidase**	**-**	**+**	**+**
**N-acétyl-beta-glucosaminidase**	**-**	**+**	**+**
alpha-mannosidase	-	-	-
**alpha-fucosidase**	**+**	**-**	**-**

Supplementary Material – Table 4. Results of the API 20E strip tests. +, positive; -, negative. Highlighted are the results that distinguish 19SA41 from *F. aquatile* DSM 1132^T and *F. xinjiangense* DSM 19743^T.

Test	Isolate 19SA41	*F. aquatile* DSM 1132^T	*F. xinjiangense* DSM 19743^T
**ONPG: test for β-galactosidase enzyme by hydrolysis of the substrate o-nitrophenyl-b-D-galactopyranoside**	**+**	**-**	**-**
ADH: decarboxylation of the amino acid arginine by arginine dihydrolase	-	-	-
LDC: decarboxylation of the amino acid lysine by lysine decarboxylase	-	-	-
ODC: decarboxylation of the amino acid ornithine by ornithine decarboxylase	-	-	-
CIT: utilization of citrate as only carbon source	+	+	+
H2S: production of hydrogen sulfide	-	-	-
URE: test for the enzyme urease	-	-	-
TDA (Tryptophan deaminase): detection of the enzyme tryptophan deaminase: Reagent- Ferric Chloride.	+	+	+
IND: Indole Test-production of indole from tryptophan by the enzyme tryptophanase . Reagent- Indole is detected by addition of Kovac's reagent.	-	-	-
**VP: the Voges-Proskauer test for the detection of acetoin (acetyl methylcarbinol) produced by fermentation of glucose by bacteria utilizing the butylene glycol pathway**	**-**	**+**	**+**
GEL: test for the production of the enzyme gelatinase which liquefies gelatin	+	+	+
GLU: fermentation of glucose (hexose sugar)	-	-	-
MAN: fermentation of mannose (hexose sugar)	-	-	-
INO: fermentation of inositol (cyclic polyalcohol)	-	-	-
SOR: fermentation of sorbitol (alcohol sugar)	-	-	-
RHA: fermentation of rhamnose (methyl pentose sugar)	-	-	-
SAC: fermentation of sucrose (disaccharide)	-	-	-
MEL: fermentation of melibiose (disaccharide)	-	-	-
AMY: fermentation of amygdalin (glycoside)	-	-	-
ARA: fermentation of arabinose (pentose sugar)	-	-	-

Supplementary Material – Table 5. Results of the API 20NE strip tests. +, positive; -, negative. Highlighted are the results that distinguish 19SA41 from *F. aquatile* DSM 1132^T and *F. xinjiangense* DSM 19743^T.

Test	Isolate 19SA41	*F. aquatile* DSM 1132^T	*F. xinjiangense* DSM 19743^T
NO3 – reduction nitrates to nitrites and nitrates to nitrogen	-	-	-
TRP – indole production	-	-	-
GLU – Fermentation of D-Glucose	-	-	-
ADH – Production of Arginine Dihydrolase	-	-	-
URE – Production of Urease	-	-	-
ESC – Hydrolysis of esculin ferric citrate	+	+	+
**GEL – Hydrolysis of gelatin**	**+**	**+**	**+**
**PNPG – Production of Beta-galactosidase**	**+**	**-**	**-**
GLU – Assimilation of Glucose	+	+	+
**ARA – Assimilation of Arabinose**	**-**	**+**	**+**
MNE – Assimilation of Mannose	+	+	+
**MAN – Assimilation of Mannitol**	**-**	**+**	**+**
NAG – Assimilation of N-Acetyl-glucosamine	+	+	+
MAL – Assimilation of Maltose	+	+	+
**GNT – Assimilation of Potassium gluconate**	**-**	**+**	**+**
**CAP – Assimilation of Capric acid**	**-**	**+**	**+**
**ADI – Assimilation of Adipic acid**	**-**	**+**	**+**
MLT – Assimilation of Malic acid	+	+	+
CIT – Assimilation of Trisodium Citrate	+	+	+
**PAC – Assimilation of Phenylacetic acid**	**-**	**+**	**+**

Supplementary Material – Table 6. Antibiotic resistance of strain 19SA41, FA and FX. +: Resistant; -, Sensitive.

Antibiotic	Dose (µg)	Isolate 19SA41	*F. aquatile* DSM 1132^T	*F. xinjiangense* DSM 19743^T
Gentamycin	30	+	+	+
Kanamycin	30	+	+	+
Rifampicin	30	+	+	+
Novobiocin	5	+	+	+
Cephalothin	30	+	+	+
Tetracyclin	30	-	-	-
Vancomycin	30	+	+	+
Oleandomycin	15	+	+	+
Lincomycin	15	+	+	+
Erythromycin	15	+	+	+
Carbenicillin	100	+	+	+

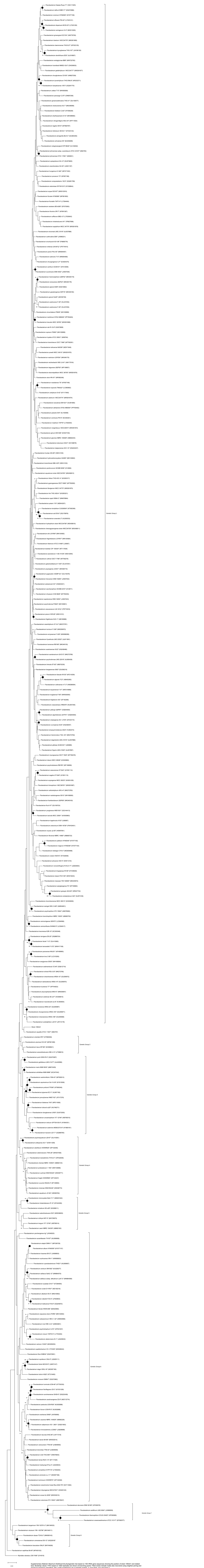

Supplementary Material: Maximum-likelihood full phylogenetic tree based on 16S rRNA gene sequences showing the position of strain 19SA41 and related taxa. Bootstrap values >70% based on 1000 replicates are shown at branching points. Filled circles indicate nodes also recovered using neighbor-joining and maximum-parsimony algorithms. Bar, 0.020 substitutions per nucleotide position. T: type strain. Your text here 3
